# Factors influencing postpartum haemorrhage detection and management and the implementation of a new postpartum haemorrhage care bundle (E-MOTIVE) in Kenya, Nigeria, and South Africa

**DOI:** 10.1186/s13012-022-01253-0

**Published:** 2023-01-11

**Authors:** Gillian Forbes, Shahinoor Akter, Suellen Miller, Hadiza Galadanci, Zahida Qureshi, Sue Fawcus, G. Justus Hofmeyr, Neil Moran, Mandisa Singata-Madliki, Faisal Dankishiya, George Gwako, Alfred Osoti, Eleanor Thomas, Ioannis Gallos, Kristie-Marie Mammoliti, Adam Devall, Arri Coomarasamy, Fernando Althabe, Lou Atkins, Meghan A. Bohren, Fabiana Lorencatto

**Affiliations:** 1 Centre for Behaviour Change, University College London, London, UK; 2 Gender and Women’s Health Unit, Centre for Health Equity, University of Melbourne School of Population and Global Health, 207 Bouverie St, Carlton, Victoria 3053 Australia; 3 Department of Obstetrics, Gynaecology, and Reproductive Sciences, School of Medicine, University of California, San Francisco, USA; 4 Africa Center of Excellence for Population Health and Policy, Bayero University Kano, Kano, Nigeria; 5 Department of Obstetrics and Gynaecology, University of Nairobi, Nairobi, Kenya; 6 Department of Obstetrics and Gynaecology, University of Cape Town, Cape Town, South Africa; 7 Department of Obstetrics and Gynaecology, University of Botswana, Gaborone, Botswana; 8 University of the Witwatersrand, Johannesburg, South Africa; 9 Walter Sisulu University, East London, South Africa; 10 KwaZulu-Natal Department of Health, University of KwaZulu-Natal, Durban, South Africa; 11 Department of Obstetrics and Gynaecology, School of Clinical Medicine, College of Health Sciences, University of KwaZulu-Natal, Durban, South Africa; 12 Effective Care Research Unit, Department of Obstetrics and Gynaecology, Universities of Witwatersrand and Fort Hare, East London, South Africa; 13 Africa Center of Excellence for Population Health and Policy, Bayero University Kano and Department of Community Medicine, Bayero University/Aminu Kano Teaching Hospital, Kano, Nigeria; 14 Tommy’s National Centre for Miscarriage Research, Institute of Metabolism and Systems Research (IMSR), WHO Collaborating Centre for Global Women’s Health Research, University of Birmingham, Mendelsohn Way, Edgbaston, Birmingham, UK; 15 WHO Collaborating Centre on Global Women’s Health, Institute of Metabolism and Systems Research, College of Medical and Dental Sciences, University of Birmingham, Birmingham, UK; 16 Department of Sexual and Reproductive Health and Research, World Health Organization, UNDP/UNFPA/UNICEF/WHO/World Bank Special Programme of Research, Development and Research Training in Human Reproduction (HRP), Geneva, Switzerland

**Keywords:** Postpartum haemorrhage (PPH), Maternal health, Low- and middle-income countries (LMICs), Clinical care bundle, Formative research, Qualitative interviews, Behaviour Change Wheel, Implementation interventions

## Abstract

**Background:**

Postpartum haemorrhage (PPH) is the leading cause of global maternal deaths, accounting for 30–50% of maternal deaths in sub-Saharan Africa. Most PPH-related deaths are preventable with timely detection and initiation of care, which may be facilitated by using a clinical care bundle. We explore influences on current PPH detection and management and on the future implementation of a new PPH bundle (E-MOTIVE) in low-resource, high-burden settings.

**Methods:**

Semi-structured qualitative interviews based on the Theoretical Domains Framework were conducted with 45 healthcare providers across nine hospitals in Nigeria, Kenya and South Africa, to identify barriers and enablers to current PPH detection and management and future implementation of a new PPH care bundle. Data were analysed using thematic and framework analysis. The Behaviour Change Wheel was used to identify potential interventions to address identified barriers and enablers.

**Results:**

Influences on current PPH detection and management fell under 12 domains: Environmental Context and Resources (drug and staff shortages), Skills (limited in-service training), Knowledge (variable understanding of the recommended practice), Behaviour Regulation (limited quality improvement culture), Beliefs about Consequences (drawbacks from inaccurate detection), Emotion (stress from the unpredictability of PPH), Social Influence (teamwork), Memory, Attention and Decision-making (limited guideline use), Social/Professional Role and Identity (role clarity), Beliefs about Capabilities (confidence in managing PPH), Reinforcement (disciplinary procedures) and Goals (PPH as a priority). Influences on bundle uptake included: Beliefs about Consequences (perceived benefits of new blood loss measurement tool), Environmental Context and Resources (high cost of drugs and new tools), Memory, Attention and Decision-making (concerns about whether bundle fits current practice), Knowledge (not understanding ‘bundled’ approach), Social Influence (acceptance by women and staff) and Intention (limited acceptance of ‘bundled' approach over existing practice). These influences were consistent across countries. Proposed interventions included: Education, Training, Modelling (core and new skills), Enablement (monitoring uptake), Persuasion (leadership role) and Environmental Restructuring (PPH emergency trolley/kit).

**Conclusions:**

A wide range of individual, socio-cultural and environmental barriers and enablers to improving PPH detection and management exist in these settings. We identified a range of interventions that could improve PPH care and the implementation of new care bundles in this context.

**Trial registration:**

ClinicalTrials.gov: NCT04341662

**Supplementary Information:**

The online version contains supplementary material available at 10.1186/s13012-022-01253-0.

Contribution to the literature

- This formative research is the first to involve healthcare providers in high-burden settings using a theoretical approach to identify the potential implementation issues of a new clinical care bundle to improve postpartum haemorrhage detection and management
- This study illustrates the application of implementation science in low-resource, high-burden settings to address a maternal health priority and implementation gap (i.e., translating postpartum haemorrhage guidelines into practice)
- This research demonstrates the benefits of considering implementation issues from the outset of project planning in order to maximise the internal validity of the trial, to ensure fidelity of bundle uptake and to optimise evaluation efforts in the future.

## Background

Almost 300,000 maternal deaths occur each year, most of which are preventable with timely access to quality maternity care when complications arise [1]. Postpartum haemorrhage (PPH) is the leading cause of maternal death, accounting for 27% of all maternal deaths worldwide and approximately 30% to 50% of maternal deaths in sub-Saharan Africa [2]. PPH is categorised as a blood loss of ≥ 500mls and severe PPH is a blood loss of ≥ 1000mls after childbirth [3]. It is typically caused by uterine atony (inadequate contraction of the uterus after birth), as well as uterine rupture, retained placental tissue, genital tract trauma, and maternal bleeding disorders [3]. PPH is largely preventable; when it does occur, early detection and prompt evidence-based management can avoid the most severe outcomes [3, 4].

Global progress in reducing PPH mortality has been slow [5], and there is an urgent need to address preventable PPH mortality and morbidity, particularly in sub-Saharan Africa, where the burden is highest. The World Health Organization (WHO) first published guidelines for the prevention and treatment of PPH in 2012 and since has regularly updated its recommendations [3, 6–9].

These recommendations include 13 clinical interventions depending on the PPH type (minor, moderate or severe), and the circumstances in which the PPH occurs (e.g., mode of birth, birth setting, timing of PPH); however, evidence of guideline implementation in practice is limited, particularly in low- and middle-income countries (LMICs) because of recognised implementation gaps in current PPH practice [10]. Also, research has shown that PPH detection is low in LMICs [11], leading to missed opportunities in providing life-saving care. In these settings, ‘visual estimation’ of blood loss is typically used to detect PPH (e.g., looking at the blood pooling on the bed under the woman or at materials used to absorb or wipe away blood); however, this approach is often unreliable and inaccurate in estimating blood loss, especially when PPH occurs [12].

Identifying effective ways of encouraging healthcare providers to change their clinical practice and follow evidence-based recommendations remains challenging [13], and the dissemination of evidence-based recommendations alone does not ensure implementation by healthcare providers [13, 14]. Failure to implement recommendations into practice can be affected by the challenges of changing well-established individual clinical behaviour, changing hospital protocols and healthcare systems [15]. One potential approach to facilitating the implementation of multi-component guideline recommendations is the introduction of a ‘care bundle’. A care bundle is a small set of evidence-based interventions (three to five) which are to be administered concurrently or in quick succession to every person with a given diagnosis. When compliance is high and all components are applied, care bundles can change clinical practice and improve patient outcomes [11, 16].

### The E-MOTIVE Research Programme

As a result, a new care bundle (‘E-MOTIVE’) for improving the first response to PPH was developed based on the evidence from the WHO's 2012 and 2017 recommendations, following a WHO Technical Consultation on PPH Bundle Development [11]. The E-MOTIVE first response to PPH bundle stands for Early detection of PPH using a calibrated drape, followed rapidly by (in no particular order): Massage of uterus, administration of Oxytocic drugs, administration of Tranexamic acid, administration of IntraVenous fluids, Examination for identifying and managing the source of bleeding, and Escalation to more advanced care, if bleeding despite first response treatment.

Implementing the E-MOTIVE bundle will necessitate a change in clinical practice behaviours. Therefore, to introduce the E-MOTIVE bundle more effectively and change behaviour, we need to first understand what factors are influencing current PPH practice (i.e., PPH detection and management) and identify a priori potential barriers and enablers to bundle uptake. This combined knowledge will help put strategies in place to hopefully target barriers and enablers of current practice that could facilitate or hinder implementation and what additional support is needed to maximise behaviour change and bundle uptake from the start, i.e. behavioural analysis prior to implementation [17]. This is particularly complex in the context of PPH, which involves two overarching behaviours comprised of multiple sub-actions: (1) PPH detection (e.g. measuring blood loss, monitoring of vital signs) and (2) PPH management (examining the woman, fetching necessary drugs, equipment and assistance, and administering drugs). Furthermore, these behaviours are often performed by multiple care providers and cadres at different time points.

**Fig. 1:**
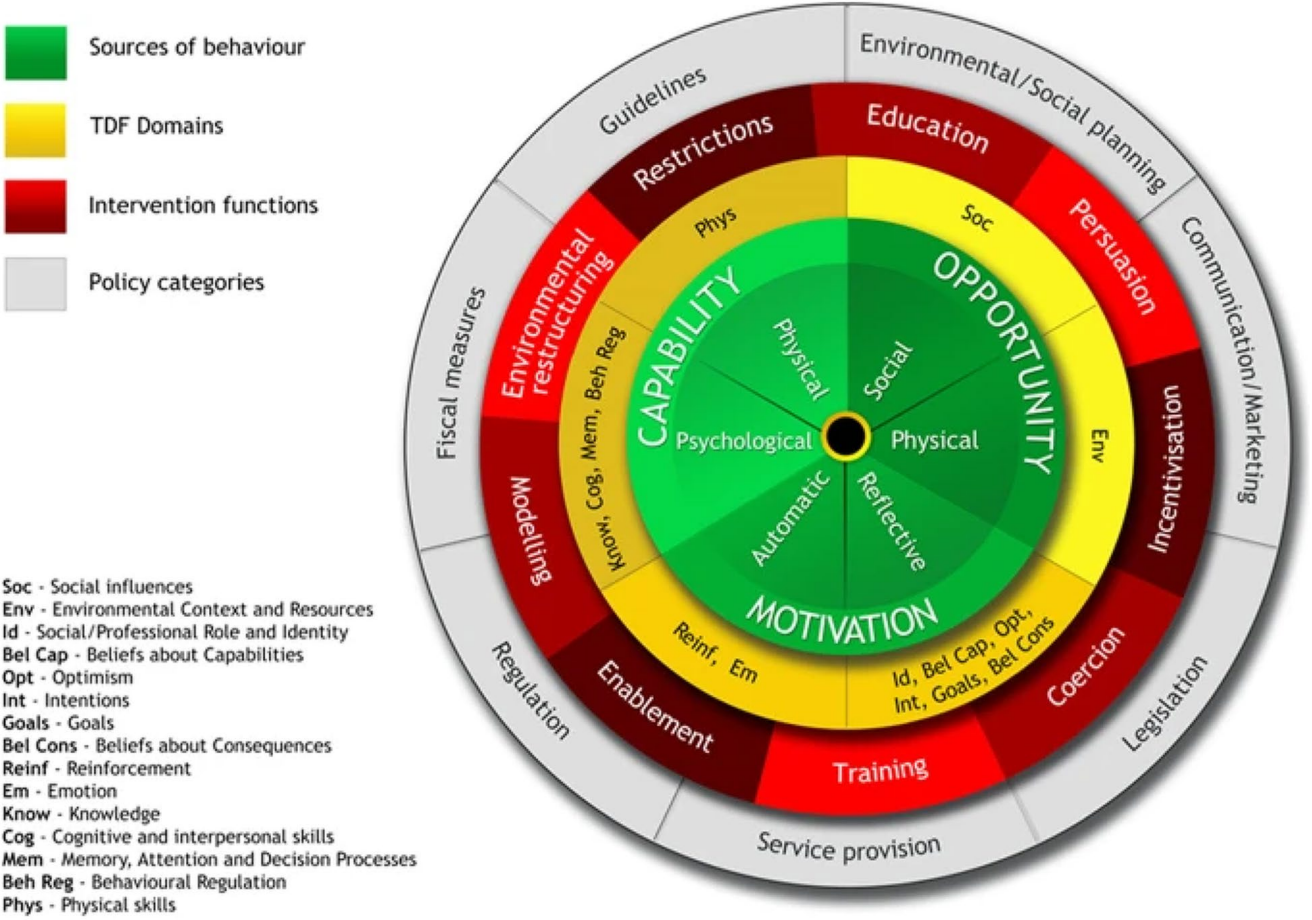
The Behaviour Change Wheel

Existing research has shown factors that influence the adherence to recommendations related to PPH detection and management in LMICs, include inaccurate estimation of blood loss after birth, limited skills to detect and to manage a PPH appropriately, and wider health system challenges, such as health workforce shortages, limited resources and training, and unreliable drug supplies [18–20]. However, existing studies did not draw on theory to guide their investigation unlike this research [19, 20]. A potential limitation of not using theory is not exploring a wide range of potential factors influencing the targeted behaviour and which implementation interventions are needed and likely to be more effective at changing behaviour. Considerable research has shown the advantages of applying behavioural theories, models and frameworks to identify and address barriers and enablers, and the uptake of evidence-based clinical care. A widely used theoretical approach for identifying what needs to change is the Theoretical Domains Framework (TDF) [21, 22]. The TDF synthesises 33 behaviour change theories into 14 domains representing cognitive, affective, social and environmental influences on behaviour. These 14 domains have also been further condensed into three over-arching minimum constructs necessary for behaviour change: Capability, Opportunity, and Motivation-Behaviour (the COM-B model) [23]. A strength of the TDF and COM-B model is that they have been mapped onto two frameworks of intervention strategies. The first is the Behaviour Change Wheel which specifies 9 broad behaviour change intervention types (i.e. education, training, incentivisation, modelling, coercion, persuasion, enablement, environmental restructuring and restriction) alongside policy categories to aid implementation (e.g. guidelines, service provision, legislation) [23] (see Fig. 1). The second, is the Behaviour Change Technique (BCT) taxonomy v1, which specifies 93 BCTs, defined as the ‘observable, replicable, and irreducible components of an intervention designed to alter or redirect causal processes that regulate behaviour’ (i.e. the ‘active ingredients;’ e.g. goal-setting, feedback on behaviour, problem-solving) [24]. There are published matrices which pair domains from the TDF and COM-B with intervention strategies in the Behaviour Change Wheel and BCT taxonomy, to suggest which types of intervention strategies are likely to be more relevant and effective in addressing different types of influences on behaviour [23, 25]. This in turn facilitates more systematic progress from initial behavioural diagnosis of barriers and enablers to selection and design of targeted, and likely more effective, implementation interventions [26]. These interlinked frameworks have been applied to identify factors influencing clinical practice and design interventions to improve implementation across a range of clinical settings including maternal care and implementation of clinical care bundles [27, 28].

In LMICs, beyond reported delays in PPH detection and inconsistent, non-evidence-based PPH management, the application of theory-based approaches to address the challenges associated with PPH detection and management-related behaviour are limited. Particularly, in sub-Saharan countries such as Nigeria, Kenya and South Africa, where there is evidence of higher proportions of maternal deaths from PPH [15–17].

Ahead of a cluster randomised trial to test the effectiveness of the E-MOTIVE bundle for the first response to PPH (ClinicalTrials.gov: NCT04341662), we aimed to identify the determinants of delayed detection and inappropriate management of PPH [27]. As part of the E-MOTIVE formative research, this study aimed to apply the TDF and the Behaviour Change Wheel to:

- Identify barriers and enablers influencing current PPH detection and management
- Identify barriers and enablers likely to facilitate or hinder the implementation of the E-MOTIVE care bundle
- Generate recommendations for potential implementation intervention types to address identified barriers and enablers and improve implementation of the E-MOTIVE bundle.

## Methods

### Design

Semi-structured qualitative interviews were conducted with healthcare providers currently working in maternity care hospitals in Nigeria, Kenya and South Africa. The full protocol has been published [29]. This study is reported according to the consolidated criteria for reporting qualitative research [30] (See Additional File 1).

### Participants

A total of 45 participants were recruited (*n*=5 participants per hospital; *n*=9 hospitals in total: 3 hospitals per country, 15 participants per country). Maximum variation sampling was used to achieve a wide range of roles including midwives, nurses, doctors, consultants and administrative/management staff of varying levels of experience and diverse settings based on hospital size and location.

### Materials

**Fig. 2:**
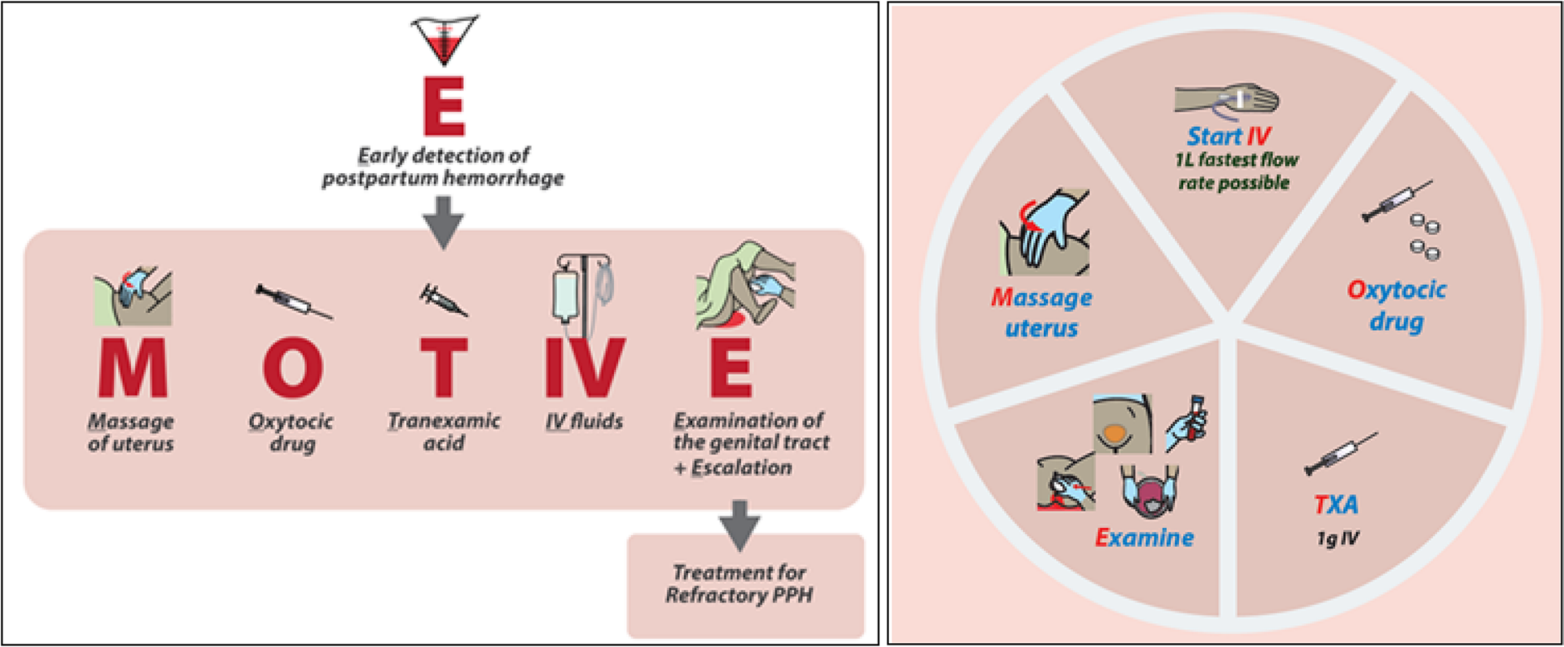
The E-MOTIVE care bundle intervention

The semi-structured guide (Additional File 2) had three broad sections exploring influences on (1) current PPH detection, (2) current PPH management and (3) potential implementation of the EMOTIVE bundle. Each section included a mixture of open questions followed by prompts structured around the domains of the TDF. For section 3, the concept of a clinical care bundle was explained, and the E-MOTIVE bundle was described, and photographs of the drape and a pictograph of the bundle elements (See Fig. 2) were shown. The topic guide was piloted in each country with midwives, nurses and doctors and refined to enhance clarity, and incorporate locally used language and definition of terms. Table 1 presents examples of TDF-based questions and follow-up prompts for each section of the interview guide.

**Table 1:** Examples of the TDF-based questions and prompts used in the interview guide (TDF domains in italics)

Section 1: PPH Detection
Q: Could you tell me a bit about your understanding of what postpartum haemorrhage (PPH) is? *(Knowledge)* Prompt: How would you define a PPH? *(Knowledge)* Q: Could you describe how is PPH typically detected in this hospital? *(Memory, Attention and Decision-making)* Prompts: What do you do? *(Social/Professional Role and Identity); *Who else is involved? *(Social Influence); *What do they do? *(Social/Professional Role and Identity); *Where (in which rooms) is PPH usually detected? *(Behaviour Regulation)*
Section 2: PPH Management
Q: Think back to the last time that a woman under your care, with a vaginal birth, had a PPH. Could you describe what happened, what did you do and why did you do it? *(Behaviour Regulation)* Prompts: How long did it take to initiate treatment? *(Memory, Attention and Decision-making);* How did you make the decision to initiate treatment? *(Memory, Attention and Decision-making); *Did you work alone or with a team? *(Social Influences); *What did you and your colleagues do to manage the PPH? *(Behaviour Regulation); *What tasks did you specifically do? *(Social/Professional Role and Identity)*
Section 3: Implementation of E-MOTIVE bundle
Q: If you and your colleagues were asked to use these treatments all together when managing PPH, as described in the bundle, what would you need to help you do it? *(Behaviour Regulation)* a. To what extent would you know what to do? *(Knowledge)* b. To what extent would you need additional skills? *(Skills)* c. To what extent do you have everything you need to perform the bundle? *(Environmental Context and Resources)* d. What do you think are the potential benefits of managing PPH using such a bundle? *(Beliefs about Consequences)* e. What do you think are the potential disadvantages of managing PPH using such a bundle? *(Beliefs about Consequences)* f. To what extent do you think managing PPH as described in the bundle, is likely to help manage PPH more effectively? *(Beliefs about Consequences)* g. How would managing a PPH as described in the bundle affect the ways you work as a team? *(Social/Professional Role and Identity)*

### Procedures

Country research teams contacted eligible healthcare providers at their place of work and provided study information sheets. After they agreed to participate, the providers signed consent forms before the interviews commenced. All interviews were conducted by experienced researchers (*n*=7: four doctors trained in maternal care, two midwives and one social scientist) from the country research teams. The interviews were conducted either face-to-face, by telephone or by Zoom due to COVID-19-related physical distancing restrictions. All interviews were audio-recorded and transcribed verbatim by the country research teams.

### Analysis

An inductive thematic and deductive framework analysis was conducted [31, 32] following three steps:

- Familiarisation of the data by the research team by listening to interview audios and reading transcripts
- *Inductive thematic analysis:* a coding matrix based on the interview guide and the research questions was used to collate the data. Data were first analysed to develop theme labels within three overarching, pre-defined categories: PPH detection, PPH management and bundle implementation. This involved identifying meaningful and important segments of text to generate descriptive summaries and grouping of similar summaries together under an inductively generated theme label. The descriptive summaries were phrased in terms of ‘belief statements’ representing a perceived barrier, enabler or mixed (i.e. both a barrier and an enabler) to current PPH practice or bundle implementation. Two researchers (GF, SA) separately produced descriptive summaries and regularly checked the summaries against theme labels for consistency. Any interpretative discrepancies were discussed until agreement was reached. Clinical accuracy of descriptive summaries and appropriateness of current practice compared to evidence-based practice was checked by SM, a midwife with expertise in PPH detection and management in LMICs. Researchers (GF, SA) checked for any contextual misunderstandings with country research interviewers
- *Deductive framework analysis:* the generated belief statements were then coded to the TDF domains they were judged to best represent. For instance, ‘a lack of accurate and objective blood measurement of blood loss leads to delays in detecting PPH’ was a barrier coded to beliefs about consequences. The mapping of belief statements to relevant TDF domains was conducted by GF and for reliability purposes, checked by FL, LA and MAB. This analysis was first done for each country. The themes generated from the analysis of transcripts from the first country (Nigeria) provided a starting point for the analysis of subsequent countries (Kenya and South Africa). Findings were then compared across countries to look for similarities and differences

### Mapping to implementation interventions

Potential strategies to address the identified barriers and enablers and facilitate implementation were identified by consulting published matrices that pair TDF domains with intervention types specified in the Behaviour Change Wheel and BCTs specified in the BCT taxonomy [24, 26, 33]. Examples of potential ways to operationalise these intervention strategies in the study context were generated from online discussions by the international, multidisciplinary research team and in-country teams.

## Results

### Participants and hospitals

Table 2 provides the sociodemographic characteristics of participants and a description of the hospitals. Forty-five healthcare providers, consisting of 14 midwives, (one male; 13 females), 6 nurses (all females), 17 doctors (9 males; 8 females), and 8 managerial staff (4 males; 4 females) participated in the study. Their overall years of experience ranged from one year to 30 years (median=8 years) and time working at their hospital from 3 months to 26 years (median=4 years). All participants currently worked in a hospital in a clinical role (*n*=37) or in a managerial role (*n*=8). The managerial staff was senior clinical staff members, with responsibility for managing and overseeing a team of clinical staff responsible for PPH detection and management (i.e. in-charge midwifery or nurse matrons or head of obstetrics). The locations of the hospitals were 2 rural and 7 urban and the burden of PPH cases was categorised as either low (less than 50 cases per year), middle (between 51 and 99 cases per year) or high (more than 100 cases per year). The median duration of the interviews was 67 min (range 38–107 min).

**Table 2:** Sociodemographic characteristics of participants and description of hospitals

Characteristics	Kenya (n=15)	Nigeria (n=15)	South Africa (n=15)	Total
**Gender**				
Female	9	7	10	26
Male	6	8	5	19
**Professional Role**				
Doctor	6	6	5	17
Midwife	6	2	6	14
Nurse	1	4	1	6
Managerial staff	2	3	3	8
Overall years of experience*				
0-5 years	NA	3	5	8
6-15 years	NA	7	5	12
16+ years	NA	5	3	8
Time at hospital*				
0-5 years	11	7	8	26
6-15 years	4	3	2	9
16+ years	0	5	NA	5
**Hospitals **				
**Location**				
Urban	3	3	1	7
Rural	-	-	2	2
**Level of burden (#PPH cases/year)**				
Low (Less than 50)	1	1	1	3
Medium (Between 51-100)	2	1	1	4
High (More than 100)	-	1	1	2

* Not all participants reported their overall experience and time at hospital (NA)

### Findings

Overall, similar influences on bundle uptake were reported from participants across countries. In the narrative summary of the results below, we highlight any country-specific influences (i.e. reported by participants from one country only) or influences for which participants from different countries had contrasting views. The findings are presented in three sections corresponding to the stated research questions. Contextual differences and similarities across the 3-country settings are discussed throughout.

### Research question (RQ)1: influences on current PPH detection and management

A total of 55 belief statements representing perceived barriers and enablers to current PPH detection and management were identified across 12 of the 14 TDF domains. No belief statements mapped to the TDF domains of optimism and intention. Table 3 presents a sub-sample of barriers and enablers identified within each TDF domain, for each country, with supporting quotes. These are summarised descriptively below, highlighting any cross-country differences and similarities within each domain. The full set of identified barriers and enablers within each domain is in Additional File 3.

**Table 3:** Summary of key barriers and enablers of PPH detection and management in Kenya, Nigeria and South Africa

Key findings grouped by TDF Domain	Freq (n=45)	**Nigeri**a	Kenya	South Africa	Example Quote(s)
**Environmental Context and Resources**					
Health hospitals organised into separate wards or rooms for maternity care	45	E	E	E	“It is made up of 2 wards, the labour room where we admit all the labour patients in, we have 5 beds in each ward” (Nurse, Nigeria)
Guidelines, clinical protocols, posters related to PPH are (not) displayed in the maternity ward	41	M	E	M	“We have SOPs in the labour ward, and they are all useful. The protocols are displayed on the walls. Everyone can check quickly”(Nurse, Kenya) “No … I don’t really know why they [posters, charts] are not displayed” (Nurse, South Africa)
Variable readiness of theatres and availability of surgeons	29	M	M	M	“We don’t have problem with them [theatres] actually, they run calls here at any point on time you have anesthetist on call, the peri-op on call, and other staffs in the theatre so we only have to inform them” (Doctor, Nigeria) “Have one theatre so there can be delays getting the patient into theatre if there is another emergency” (Doctor, South Africa)
**Skills**					
Inconsistent in-service training in PPH	44	M	M	M	“We do patient training, for example sometimes, at a time, there was a time when there was a demonstration on the use of anti-shock garment and all that, and most of the training are not online” (Doctor, Nigeria) “There is not any other training outside medical school and residency training program specifically on postpartum haemorrhage” (Admin, Nigeria)
Regular continuing medical education (CME), including case reviews, will reduce PPH cases	16	E	E	E	“We also undertake CMEs, monthly or twice a month” (Midwife, Kenya)
Continuous and additional training helps to keep up-to-date with new approaches and current recommendations	14	X	E	E	“It is very much helpful because you cannot only rely on the knowledge from training school while there are new trends” (Midwife, Kenya)
**Knowledge **					
Variable understanding of how to detect different aetiologies of PPH	44	E	M	B	“Postpartum haemorrhage is by definition, a vaginal bleeding after birth. And in terms of quantification when a woman has lost more, more or equal 500 ml of blood at the definition” (Doctor, South Africa) “I think it is not having enough knowledge [among new staff] on the PPH…Because maybe sometimes we are unable to quantify the amount of blood lost, then we say this is PPH, or this is just normal blood loss.” [Doctor, Kenya]
Variable understanding of what constitutes appropriate clinical practice for PPH management	37	E	M	B	“We always have them be made aware of these guidelines. But for the rest of us, we [Doctors] sort of go ahead without knowing or reading the guidelines” (Head of Dept, South Africa) “We always use them as a reference. We want all of us to have the same level of understanding and care” (Midwife, South Africa)
Higher-level hospitals receive limited information about the previous care received by woman referred from lower hospitals	23	B	B	X	“In some cases, the patient arrives here with no IV line, no samples, and quite often the reason for referral could be something they could have easily managed, such as retained placenta, but they do not know what to do” (Nurse, Kenya)
**Behavioural Regulation**					
Receiving feedback on current practice helps identify areas for improvement	38	E	E	E	“I think anything that could make it better, it’s still welcomed” (Doctor, Nigeria)
Lack of quality improvement strategies for PPH (e.g., meetings where feedback is given)	17	B	B	B	“We would like to hold such meetings more frequently. Sometimes due to inadequate staffing we skip monthly meetings” (Midwife, Kenya)
Current strategies in place, e.g., having a PPH kit, continuing education and hospital specific protocols are perceived to work	16	X	E	E	“I am not aware of any specific strategies of improvement, apart from perhaps resuming CMEs, whereby clinicians would continue to get training on PPH management” (Doctor, Kenya)
**Beliefs about Consequences **					
Maternal mortality due to PPH is low if birth occurs within hospital	38	B	M	M	“It is really it’s a big issue especially with those that are referred from another hospital or referred from home...… [if] it occurs in our centre, because of early intervention and prompt treatment, we usually have good outcome” (Doctor, Nigeria) “It’s very common we see it very often, but I think we respond to it quite quickly .... within an hour or two after birth…. so, every PPH will get picked up at a certain” (Midwife, South Africa)
Lack of accurate and objective measurement of blood loss delays detection of PPH	34	B	B	B	“We still rely on the gross method that we use, of assessing the amount of blood based on the amount of blood by our under-pads or the sanitary pads that we use or occasionally if it is too much, we use the kidney dish to collect the blood, but you know we have a high tendency for underestimating because it will not take into account” (Admin, Nigeria)
Referrals by other hospitals can be too late for the receiving hospital to effectively manage the PPH	21	B	B	B	“Late presentation is also one of the issues and challenges that we have especially from those who are coming from outside because most times they would have gone to other peripheral hospitals” (Doctor, Nigeria)
**Emotion **					
Negative emotions resulting from the unpredictability and fatality associated with PPH	40	M	M	M	“You are a little bit on edge you know that this is a complication it’s a leading cause of maternal mortality” (Midwife, South Africa) “it’s an incident that can be managed. So, it’s just that nothing to fear or to worry about” (Doctor, Nigeria)
Limited impact from emotions as doctors’ work ‘on autopilot’	7	E	E	X	“At that time, you are being put on one autopilot that all you're trying to do is trying to save that person’s life, so even thinking of yourself as being stressed out does not even come into play, it’s after you’ve finished” (Doctor, Nigeria)
Emotional support is received from colleagues and religious beliefs	7	E	E	X	“There is need for debriefs, especially after a complication ends in maternal death” (Midwife, Nigeria)
**Social Influences **					
Team-working is necessary to manage a PPH	42	E	E	E	“We worked with the midwife who conducted the delivery and also some other nurses and me as a Medical Officer” (Doctor, Kenya)
Team working reassures individuals that support is readily available	8	E	E	E	“We work well as a team and when you call for the help everyone comes here. Everyone comes here, everyone doctors, nurses, even the cleaners at times” (Midwife, South Africa)
Mothers lack knowledge of PPH signs and symptoms can delay timely detection and management of PPH	3	X	X	B	“I’m bleeding too much, so you educate your patient please call for help as much as bleeding after delivery is normal but excessive bleeding is not normal” (Midwife, South Africa)
**Memory, Attention and Decision-making**					
International guidelines adapted into hospital-specific clinical guidelines are more likely to be implemented by staff	36	M	M	M	“We use WHO guidelines. But we have tailored these to the basics. We sometimes substitute the drugs of choice as per the guidelines, with what we have available in the hospital” (Doctor, Kenya) “We [Doctors] sort of go ahead without knowing or reading the guidelines” (Head of Dept, South Africa)
Good clinical understanding of when and how to escalate to refractory PPH management intervention	24	M	E	M	“Patient can be taken to a theatre…. under resuscitation on IV fluids and blood if available, check for cervical laceration, and then since the uterus is flabby has not contracted, we can institute other modalities like minimally invasive modalities or more extensive modalities” (Doctor, Nigeria) “Oxytocic was given, then checking if the uterus has contracted yet and then he noticed that the uterus was not contracting and he did the bimanual contraction of the uterus until the doctor came” (Midwife, South Africa)
Delayed treatment due to reliance on vital signs to detect a PPH	10	M	E	M	“Especially from the signs and symptoms the mother starts experiencing headaches, light headedness and also dizziness and from the vitals when you check the blood pressure” (Doctor, Kenya) “The extent of blood loss is the first thing that we pay attention to, and then the patient’s vital status” (Doctor, Nigeria)
**Social/Professional Role and Identity **					
Good levels of role clarity	34	E	E	E	“My role is that after I detect that the woman has PPH, I will call the attention of my colleague and later on the doctor on call” (Midwife, Nigeria)
There is a clear division of tasks based on profession	33	E	E	B	“The person doing the delivery, the midwife will examine and then they would call, usually call the doctor who take over examination or just double check” (Midwife, South Africa)
The team managing a PPH should be multi-disciplinary	10	X	E	X	“Midwives even the sub-staff can help but if there are many doctors on ground they can come and help in managing the patient. For instance, one setting the line, one taking the sample for blood for grouping and cross-matching or some applying anti-shock garment, so it is multi-disciplinary activity” (Doctor, Nigeria)
**Beliefs about Capabilities **					
Varied levels of confidence in ability to detect and manage PPH	24	M	M	M	“I can confidently say I can be able to do it” (Midwife, South Africa) “Not all workers, like nurses and intern doctors [are confident]” (Doctor, Kenya)
Concerns about abilities to detect and manage PPH with un-booked women	6	B	X	X	“The patients come from outside (un-booked] because that's where the problem is” (Admin, South Africa)
Coping with the quick and sudden onset of PPH can be challenging	2	B	X	X	“Her condition suddenly changed, and she showed symptoms of PPH” (Midwife, Kenya)
**Reinforcement **					
Varied approaches to disciplinary procedures across hospitals and countries	29	E	B	B	“When something goes wrong, the entire team should take responsibility, review what happened and use it positively as a learning experience” (Doctor, Kenya) “If there was a higher incident of obstetric haemorrhage ……they would either give training or they would discipline the people involved” (Midwife, South Africa)
Disciplinary procedures across hospitals and countries are unlikely to change PPH care	18	B	B	X	“The most important thing is about having the passion…. [to] save the patient rather than thinking that whether you are going to be disciplined for doing it or not” (Admin, Nigeria)
**Goals **					
Mortality associated with PPH makes it a clinical priority	42	E	E	E	“It’s a very huge priority because for me it’s a very serious challenge and very serious priority that I need to consider it with too much seriousness. Because it’s something that I may just…can result to maternal mortality within the blink of an eye” (Midwife, Nigeria)
Eclampsia perceived as a higher priority than PPH because eclampsia causes more mortality	3	B	B	B	“Severe pre-eclampsia and eclampsia are first on priority list as they cause more mortality” (Doctor, Nigeria)

This table presents a sub-sample of belief statements and the overall frequency count of the number of participants who expressed the belief (n=maximum of 45 participants). Belief statements are classified as either a barrier (B), Enabler (E) or Mixed (M) for each country

X=belief statement not identified in the data collected from the country

### Environmental context and resources

The influence of environmental context and resources was mixed across all countries. Provision of PPH care was facilitated by all hospitals being organised into separate wards or rooms for maternity care and by having guidelines, protocols, posters and/or charts related to PPH care displayed in some maternity wards across all countries. A common barrier was inconsistent supplies of drugs, particularly in Nigeria where women or their families often had to procure tranexamic acid themselves. Other barriers were delays in transporting women referred from lower-level hospitals to higher-level hospitals because of a lack of ambulances (all countries), unreliable blood supplies (only in Nigeria and South Africa), staff shortages to manage PPH emergencies (all countries) and lack of tools and equipment to measure blood loss (only in Nigeria and Kenya).

### Skills

The influence of skills related to PPH detection and management was also mixed across all countries. Training on PPH received since midwifery, nursing or medical school was inconsistent, with some healthcare providers having received in-service training whilst others had no additional training since pre-service certification. Continuous training was seen as an enabler to keeping updated with new approaches and was recommended by participants in Kenya and South Africa. Participants reported more training for inexperienced or new staff was required in Kenya and Nigeria.

### Knowledge

Participants across countries reported differing knowledge of definitions of PPH, different knowledge of guidelines, differing awareness of the signs and symptoms of a PPH and different steps constituting appropriate PPH management. These findings were often in contradiction to WHO guidelines. Participants reported recognising PPH as an obstetric emergency and felt that they were knowledgeable at appropriately detecting and managing PPH.

### Behaviour regulation

For all countries, a key enabler was receiving feedback on current practice because it helps identify areas for improvement and in Kenya and South Africa existing strategies such as continuing education, hospital-specific protocols and having a PPH emergency kit reportedly helped to promote appropriate PPH care provision. In Kenya and Nigeria, a lack of information sharing between hospitals when referring women was a relevant hindrance to PPH management and monitoring of PPH clinical practice using audit and feedback was reported to be limited in all countries.

### Beliefs about consequences

Despite PPH being the largest contributor to maternal mortality and described as a ‘common’ and ‘big issue’, participants in this study did not always perceive it to be as serious a concern compared to other maternal health issues. For all countries, negative consequences of current practice were delays in PPH detection because of a lack of an appropriate and objective measure of blood loss and late referrals from other hospitals to effectively manage PPH.

### Social/professional role and identity

Midwives and nurses were most often the first responders to PPH, responsible for deciding when to call for help after observing a significant blood loss or a sudden onset of bleeding after birth in all countries. Midwives and doctors performed examinations to determine the cause of the blood loss and took vital signs to detect and confirm a PPH. Therefore, an important enabler was a good level of role clarity (individual and team) in all countries, division of tasks based on clinical role (in Nigeria and Kenya) and having a multi-disciplinary team managing a PPH (only reported in Kenya).

### Emotion

Healthcare workers, particularly first responders, reported an association between some negative emotions such as panic and stress, they felt when managing a PPH with the unpredictability and potential for maternal mortality from PPH.

### Social influence

The impact of social influence was mixed across countries. An enabler for some participants was teamwork, which they described could speed up and improve the management of PPH; therefore, it is necessary to appropriately manage PPH. In contrast, some participants believed PPH had to be detected and managed alone in some hospitals often because of staff shortages that were reported across all countries.

### Memory, attention and decision-making

Memory, attention and decision-making influences were also mixed. In all countries, more attention was given to using guidelines to inform clinical care if international guidelines were adapted into hospital-based clinical guidelines. A common barrier in South Africa was that more consideration was given to vital signs (observing a decreasing blood pressure and an increasing pulse rate) than blood loss to detect a PPH, which may delay treatment. Participants often reported detecting PPH using visual blood loss estimation methods, such as looking at the amount of blood on linen or on the floor, using kidney dishes to collect blood or counting the number of blood-soaked linens or swabs. Decisions made during this period of diagnosis were sometimes unlikely to include any immediate treatment to manage the PPH. After diagnosing a PPH, management sometimes followed a 'wait and see’ approach, where clinicians would implement one treatment measure at a time and wait to see if the woman responded to it. The treatments mentioned as the primary or first-line treatments included uterine massage, oxytocic drugs (e.g. oxytocin and misoprostol) and IV fluids. Some of the reported current care conflicted with the evidence-based practice for PPH detection and management, for example not administering the dosage of oxytocin recommended by WHO guidelines.

### Beliefs about capabilities

Beliefs about capabilities were a mixed influence, with healthcare providers reporting varying levels of confidence in detecting and managing PPH. In Nigeria, specific barriers were the need to cope with the rapid onset of PPH and abilities to detect and manage PPH with a woman not booked for birth in the hospital, because of having no details of the medical history of potential high-risk factors for PPH (compared to a woman booked to give birth in the hospital, who therefore has a medical history obtained).

### Reinforcement

There were varying approaches to disciplinary procedures for the mismanagement of PPH across hospitals and countries. In Nigeria and Kenya, disciplinary procedures were considered a barrier to PPH detection and management; therefore, disciplinary actions were not required or recommended with more training being preferrable. One participant in South Africa expressed fears of punishment (i.e. disciplinary action taken) for failing to manage a PPH appropriately.

### Goals

Across all countries, an enabler was that PPH was perceived to be of high importance because it can cause maternal deaths if not detected and managed appropriately.

### RQ2: Potential influences on the future use of the E-MOTIVE bundle

Thirty-eight belief statements representing barriers and enablers to implementing the E-MOTIVE bundle were identified across nine TDF domains. No beliefs were mapped to the TDF domains of Behaviour Regulation, Optimism, Reinforcement, Goals and Emotion. Table 4 shows a full list of belief statements across TDF domains, classified as a barrier, an enabler or mixed across the three countries, alongside supporting quotes.

**Table 4:** List of TDF domain belief statements about factors potentially influencing uptake of the E-MOTIVE bundle In Kenya, Nigeria and South Africa

Key findings grouped by TDF Domain	E-MOTIVE Component	Freq (n=45)	Nigeria	Kenya	South Africa	Example Quote(s)
**Environmental Context & Resources **						
Concerns about cost and availability of the calibrated drape	Calibrated Drape	13	B	B	B	“This item looks expensive. I am imagining if it we discard the blood and sterilize the tool could be used by another, and therefore reduce the cost” (Midwife, Kenya)
Use of drape requires appropriate beds	Calibrated Drape	5	B	B	B	“The funnel won’t be able to hang down so then, I think it’s gonna make it difficult for them to detect the blood loss cause if it’s not hanging it’s gonna be flat on the bed” (Midwife, South Africa)
Mixed views about the supply chain and ready availability of oxytocin	Oxytocic drugs	15	M	M	M	“They [oxytocic drugs] are usually kept at the store at the pharmacies so they are not…even the cold chain has been broken” (Doctor, Nigeria) “I think it’s stored in the fridge, ... so anytime, it’s always there” (Doctor, Nigeria)
Poor quality brands of oxytocin procured	Oxytocic drugs	6	B	B	x	“Particular brand of oxytocin that we got in recent times which we have actually reported that we thought it wasn’t quite effective” (Admin, Nigeria)
Limited tranexamic acid supplies due to high costs	Tranexamic acid	7	B	B	x	“They [hospital] don’t supply it... [available] in the pharmacy, and it is costly.... some patients cannot afford to buy it” (Midwife, Nigeria)
IV fluids readily available in the ward	IV Fluids	12	E	E	E	“It is easy because we have it in the emergency drugs” (Nurse, Nigeria)
More than one person is required to do an examination (Lack of staff)	Examination	6	M	M	M	“It is not something you need to do alone, you need about two assistants to help you” (Doctor, Nigeria) “As a midwife, I examine the woman” (Midwife, Kenya)
Inadequate lighting due to electricity outages limits ability to conduct examinations	Examination	5	B	B	B	“There are periods that this light has had a problem and then the generator also had an issue” (Admin, Nigeria)
More staff needed to deliver the E-MOTIVE bundle	All components	2	B	B	X	“You’ll find yourself sometimes alone you understand manpower the other one has gone to receive a baby, you will be left with the patient, so you just have to do it alone” (Midwife, Nigeria)
**Skills and Knowledge **						
Lack of education and skills on how and when to use a calibrated drape	Calibrated Drape	28	B	B	B	“People need to be educated on how to work with this, they need to know how to use it first, and then they need to practice” (Doctor, Nigeria)
Training to use tranexamic acid is required, particularly for midwives and nurses who do not currently administer it	Tranexamic acid	8	B	B	B	“It is not specifically ordered by the doctor, when we are managing the patient, we do not think of this Cyclokapron. We only think of it when the doctor prescribes it” (Midwife, South Africa)
Further training on how to use the E-MOTIVE bundle is required	All components	22	M	M	M	“The team should be trained [on the bundle components] because all these treatments need extra hands. Therefore, everyone should be well acquainted to assist when required” (Doctor, Kenya)
**Beliefs about Consequences**						
Accurately measuring blood loss improves detection of PPH	Calibrated Drape	45	E	E	E	“One of the biggest challenges estimating getting a close to a good estimate of the blood loss so if you are able to get something that gives that then I think it would be a very good thing” (Midwife, Kenya)
Uterine massage is effective in managing PPH when used in conjunction with Oxytocics/ uterotonics	Massage	16	E	E	E	“I start massaging I will now inject oxytocin but immediately you start massaging you will see the uterus starting to contract…. definitely it comes with reduction in blood loss” (Doctor, Nigeria)
Oxytocin is routinely administered as it is effective at preventing and managing PPH	Oxytocic drugs	15	E	E	E	“Usually by the time you give oxytocin, the PPH often stops, other than in a few cases” (Midwife, Kenya)
Beliefs that using the bundle is limited (by not having tranexamic acid readily available)	Tranexamic acid	10	B	B	B	“Sometimes it is not available in the nearby pharmacy we have” (Midwife, Nigeria)
Tranexamic acid is effective at stopping bleeding after oxytocic drugs have not worked, although tranexamic acid is not more effective than oxytocin	Tranexamic acid	9	M	M	M	“You only use oxytocin, and they respond so there is no need for you to give Cyclokapron [tranexamic acid] and other drugs in the bundle” (Midwife, South Africa) “Tranexamic acid is there to help clot the blood, so I don't know if that makes a difference that I think oxytocin is number one” (Head of Dept, South Africa)
IV fluids help stabilise the patient by maintaining haemodynamic status	IV fluids	6	E	E	E	“…. we have delays in getting blood so initially we have to make do with IV fluids until the blood is available to maintain haemodynamic status” (Doctor, Nigeria)
Using the bundle will increase the effectiveness and improve current management of PPH	All components	34	E	E	E	“…it [the bundle] is going to improve the services rendered and then it will really reduce the morbidity and mortality associated with this condition” (Doctor, Nigeria)
Using the bundle will reduce PPH mortality and morbidity	All components	10	E	E	E	“It [the bundle] will impact positively because it will reduce morbidity and mortality associated with postpartum haemorrhage” (Manager, Nigeria)
Concerns about misoprostol not being part of the bundle when it is perceived to be effective at treating a PPH	All components	1	x	x	B	“We use misoprostol as well before tranexamic acid” (Head of Dept, South Africa)
**Social Influences **						
Concerns about giving uterine massage causing discomfort and pain to women	Massage	4	B	B	X	“Some patients will refuse” (Midwife, Nigeria)
Some women refuse to have an examination of the genital tract	Examination	13	B	B	B	“Sometimes the mother becomes uncooperative. It is uncomfortable for mothers so some refuse to be examined” (Midwife, Kenya)
**Memory, Attention and Decision-making **						
Concerns about calibrated drape fitting in with current methods for estimating blood loss	Calibrated Drape	4	B	B	B	“I think it’s gonna make it difficult for them to detect the blood loss cause if it’s not hanging it’s gonna be flat on the bed” (Midwife, South Africa)
Uterine massage currently used as first line treatment for PPH	Massage	38	E	E	E	“…… know to rub the uterus, that one they definitely have to rub the uterus and then the drugs” (Doctor, South Africa)
Oxytocin currently administered simultaneously with uterine massage	Oxytocic drugs	38	E	E	E	“Uterine massage and oxytocics are quite important because of their significance in preventing postpartum haemorrhage” (Admin, Nigeria)
Tranexamic acid is not routinely used (unless as a last resort when oxytocin has not worked)	Tranexamic acid	38	B	B	B	“Normally we give tranexamic when oxytocin fails, when we do all the measures and it fails then we get a tranexamic acid” (Midwife, Nigeria)
IV fluids are routinely used managing PPH	IV Fluids	21	E	E	E	“That is the second thing they will do. So, we use it always” (Nurse, Kenya)
Genital tract examination currently done to detect cause of bleeding	Examination	45	E	E	E	“We do it [examine] immediately after delivery to know whether there is any tear, and if the patient is also bleeding, we also still assess” (Nurse, Nigeria)
Current PPH management already includes all individual components of E-MOTIVE, but not delivered as a care bundle	All components	9	B	B	B	“I think it is better to use one after the other, than using all at once. The reason for the bleeding, could be trauma, tears, so when you repair a trauma the bleeding stops” (Midwife, Kenya)
**Social/Professional Role and Identity **						
Mixed beliefs that both doctors and midwives or nurses can do examinations	Examination	19	M	M	M	“It should be…the registrar does it [examine], and the senior registrar and then maybe the midwife” (Doctor, Nigeria) “As a midwife, I examine the woman” (Midwife, Kenya)
Teamwork is required to use the E-MOTIVE bundle where each person will have assigned role(s)	All components	1	X	E	X	“The bundle actually emphasizes and enhances teamwork” (Doctor, Kenya)
**Beliefs about Capabilities **						
Confident in ability to give a uterine massage	Massage	9	E	E	E	“All the staff should be confident in performing a uterine massage” (Admin, Nigeria)
Confident in administering oxytocin	Oxytocic drugs	10	E	E	E	“So, I can confidently say I can be able to do it” (Midwife, South Africa)
Healthcare providers are confident at using IV fluids	IV fluids	5	E	E	E	“100% [confident], It works. It is very effective” (Midwife, Nigeria)
Clinical staff feel (un-)skilled and (not) confident in examining the genital tract	Examination	9	M	M	M	“If there is something I find that I am not sure about I can always ask my seniors, midwife or the medical officer on duty” (Midwife, Kenya) “All the doctors can effectively do examination, even a significant number of our midwives can be able to do [it]” (Admin, Nigeria)
Lack of confidence will affect overall use of the E-MOTIVE bundle	All components	6	B	B	X	“Not everyone is confident, newer staff might not manage all of them all at once” (Nurse, Kenya)
**Intentions **						
Potential lack of buy-in of using the bundle over existing practice	All components	3	B	B	B	“We massage the uterus, administer oxytocic, tranexamic acid, and IV and also examine the vaginal track to remove any retained tissues. We do not refer to it as bundle, but we do all these in the EMOTIVE bundle” (Midwife, South Africa)

This table presents all belief statements and the overall frequency count of the number of participants who expressed the belief (n=maximum of 45 participants). Belief statements are classified as either a barrier (B), enabler (E) or mixed (M) for each country

X=belief statement not identified in the data collected from the country

### Beliefs about consequences

Collectively, the feedback received about introducing the E-MOTIVE bundle to clinical practice was positive across all countries. Many participants said that using the bundle had the potential to improve current PPH management and to reduce PPH morbidity and mortality in their hospitals or country. The perceived benefits of using the bundle were the accurate measurement of blood loss which could increase the effectiveness of PPH detection and consequently improve PPH management and reduce PPH mortality and morbidity. The bundled approach of using massage and oxytocin together was thought to be advantageous, although not including misoprostol as part of the bundle was a potential disadvantage because misoprostol is typically used in conjunction with oxytocin, especially where the latter was thought to have poor quality. Potential barriers were how the drape may limit women to only recumbent birth positions (lying flat on their back) and that counting of swabs placed in the drape may be time-consuming.

### Environmental context and resources

The influence of environmental context and resources was mixed. An enabler was having a plentiful supply of oxytocin and IV fluids and having a large enough team to deliver the bundle as intended. A key barrier to bundle implementation was the limited availability of tranexamic acid in the maternity ward and in the wider hospital. Other barriers were an inadequate supply of and anticipated high ongoing cost of calibrated drapes, having poor quality oxytocin, bed type not suitable for using the calibrated drape, hospital prone to power cuts that could impede internal examinations and staff shortages.

### Memory, attention and decision-making

Memory, attention and decision-making was an enabler because components of the bundle were already used independently, such as examinations to detect the cause of bleeding, uterine massage as the first-line treatment for PPH and oxytocin currently administered simultaneously with the uterine massage. An important barrier was that tranexamic acid was not routinely used (unless as a last resort when oxytocin had not worked). Despite an overall positive evaluation of the bundle, and a recognition that the majority of bundle elements were already used in current practice, there were concerns raised about how collecting blood loss in a calibrated drape would fit in with current practice.

### Beliefs about capabilities

Confidence in performing different components of the bundle varied. Across all countries, participants reported feeling confident in their capability to give oxytocin, IV fluids and uterine massage, but there was varying confidence in examining the genital tract and this also varied across cadre, e.g. doctors were often more confident than midwives. Participants also did not feel confident in using a bundled approach and in giving tranexamic acid, specifically in Nigeria and Kenya where tranexamic acid was not routinely used for PPH management.

### Skills and knowledge

Both skills and knowledge were reported to be a barrier to implementing the bundle, particularly a lack of education and skills around how to use components of the bundle that are not routinely used in current practice (e.g., a calibrated drape, tranexamic acid). A further barrier was a lack of understanding of the concept of a clinical care bundle and a need for further training on using a bundle approach.

### Social/professional role and identity

There were mixed views about whether certain professional groups would be allowed to perform certain components of the bundle, for instance, whether in addition to doctors, midwives or nurses could perform genital tract examinations. A potential enabler of the overall use of the bundle was assigning roles and a need to work as a team.

### Social influences

Social influence was a barrier to the massage and examination components of the bundle. Some women refuse to have an internal examination and staff have concerns about causing discomfort and pain to a woman when applying a uterine massage.

### Intention

The influence of intention is a potential barrier, with reported concerns over buy-in (i.e. failure to see benefits) to the bundle approach over existing practice, because the majority of bundle elements are already being practised—albeit independently rather than bundled.

Overall, similar influences likely to facilitate or hinder the implementation of the E-MOTIVE care bundle were consistently found across all countries.

### RQ3: Mapping of identified barriers and enablers to implementation interventions

Table 5 presents a mapping of identified barriers and enablers to potential intervention strategies to address these and improve current practice and maximise future bundle implementation. We proposed 5 implementation interventions to address the identified barriers and enablers: Calibrated Drape, E-MOTIVE training, E-MOTIVE champions, PPH emergency trolley/kit and Audit and Feedback. These interventions focus on increasing the competencies and motivation of staff to adopt E-MOTIVE and to address existing contextual and socio-cultural factors which could hinder the uptake and optimal delivery of E-MOTIVE. One strategy which is part of the E-MOTIVE bundle is a new blood collection measurement tool (i.e., a calibrated drape) that targets delays in PPH detection and inaccurate measurement of blood loss. Therefore, this requires specific training on how to use this new calibrated drape appropriately and includes broader education on the purpose of clinical care bundles, such as E-MOTIVE, and on recommended PPH management to address the identified areas of sub-optimal PPH care. Other barriers addressed by training include administering tranexamic acid, which is rarely used to treat mild PPH in Nigeria and Kenya and only prescribed by doctors in South Africa, inadequate team communication skills, breaking down any hierarchical clinical practices and reassuring midwives of their enhanced role in administering tranexamic acid where there is no specific local hospital protocol specifying that nursing staff can administer tranexamic acid.

**Table 5:** Identified barriers and enablers mapped to proposed implementation interventions using the BCW

**COM-B **Model*	**TDFDomain**	**Barriers and enablers to improving current PPH detection and management and implementing E-MOTIVE **	**Intervention Type**	**Behaviour Change Techniques**	**Proposed Implementation Interventions **
**Capabilities (Physical and Psychological)**	Skills	Inconsistent ongoing training on PPH; lacking in some core skills (Barrier)	Training, Education, Modelling, Enablement	Demonstration of the behaviour; Instruction on how to perform a behaviour; Behavioural rehearsal/practice; Habit formation; Action planning; Feedback on behaviour; Feedback on outcome of behaviour; Salience of consequences	- Training to provide core or new skills required to deliver the bundle; for example, using new calibrated drape, administering tranexamic acid by midwives Additional education and training to improve knowledge of PPH detection and management and an understanding of a bundled approach of care (i.e., use all components of the bundle), provide setting specific guidelines - Introduce audit and feedback as a way to monitor bundle uptake and to prompt implementation by providing feedback to staff; set targets
	Knowledge	Varying understanding of PPH aetiologies including signs and symptoms (Mixed)			
	Behaviour Regulation	Acknowledged missed opportunities to improve current PPH practice (Enabler)			
	Memory, Attention & Decision-making	Visual estimation of blood loss and deciding to use the bundle and to use the bundle as intended (Barrier)			
**Opportunity (Physical and Social)**	Environmental Context and Resources	Varying accessibility of stock of consumables, equipment and drugs (Mixed) More staff is needed to deliver the bundle (Barrier)	Enablement, Environmental Restructuring, Training, Modelling	Prompts/Cues; Adding objects to the environment; Credible source; Social support (Practical); Social support (Emotional)	- Introduce a trolley/kit to organise stock of consumables, equipment and drugs for PPH into one place to reduce time taken to fetch everything; reduce the sense of panic felt by staff by sudden and unpredictable onset of PPH
	Social Influence	Teamwork is required to deliver the bundle (Barrier)			
**Motivation (Automatic and Reflective)**	Beliefs about Capabilities	Relying on subjective estimation of blood loss leading to delayed or inaccurate detection of PPH (Barrier)	Enablement, Environmental Restructuring, Persuasion, Modelling, Education, Training	Demonstration of behaviour; Behavioural rehearsal/ practice; Pros and cons; Information on health consequences; Information on emotional consequences; Verbal persuasion to boost self-efficacy; Social support (Practical); Social support (Emotional); Social support (Unspecified)	- Calibrated drape as a new tool to both prompt and facilitate detection; provide more accurate blood loss - Introduce a bundle champion in a leadership role who can encourage, support and model bundle use; promote collective use of the bundle as a means to motivate staff to adopt new clinical practices or adapt existing practice and to overcome any resistance to change
	Beliefs about Consequences	Beliefs that quicker and accurate PPH detection can reduce maternal mortality (Enabler)varying confidence in delivering some elements of the bundle (Enabler)			
	Goals	Reducing PPH is important; it is main cause of maternal fatalities (Enabler)			
	Social/Professional Role and Identity	Mixed views on both doctors and midwives or nurses performing some or all parts of the bundle (Mixed)			
	Intention	Potential lack of buy-in to the bundle over existing practice (Barrier)			
	Emotion	Coping with negative feelings of panic and stress from unpredictably of PPH (Barrier)			

*COM-B Model (C=Capability; O=Opportunity; M=Motivation and B=Behaviour) Michie et al. 2011

Other BCW intervention types considered unacceptable included Incentivisation, Coercion and Restriction

Other interventions operate on a day-to-day basis to target any resistance to adopting E-MOTIVE by providing on-site leadership (e.g., E-MOTIVE champions) support to encourage and to demonstrate using the bundle. Also, to address the stress and anxiety often felt by staff when coping with a PPH, in particular in sourcing all necessary drugs and equipment by introducing a regularly stocked PPH-specific emergency trolley/kit to keep everything readily available in one place. At the hospital level, healthcare providers were encouraged to routinely use E-MOTIVE by monitoring uptake and subsequently give feedback to identify areas for further improvements (i.e., Audit and Feedback).

## Discussion

This study aimed to understand what factors influenced PPH detection and management, a global health priority with demonstrated evidence of guideline implementation challenges in current practice, and to identify a priori what it would take to implement a newly developed clinical care bundle that aims to increase adherence to evidence-based practice. The E-MOTIVE project’s overall goal is to reduce PPH-related maternal morbidity and mortality through improved early detection and management of PPH.

We identified a wide range of influences on PPH detection and management that could affect the future delivery of the new E-MOTIVE bundle. Drawing on the TDF, the thirteen domains to address during implementation were Skills; Knowledge; Behaviour Regulation; Memory, Attention and Decision-making; Environmental Context and Resources; Social Influences; Beliefs about Consequences; Beliefs about Capabilities; Social/Professional Role and Identity; Emotion; Reinforcement, Intention and Goals. There were multiple barriers including divergence in participants’ knowledge, insufficient skills training and poor teamwork, based on professional hierarchies. Contextual factors were a lack of supplies and staff shortages. These are consistent with the findings from other research investigating what influences PPH care [18–20].

Despite the heterogeneous sample, there was consensus across some beliefs and there were areas of disagreement where beliefs that were barriers in one country were enablers in another. At an individual level, similar beliefs seemed to influence current PPH care practices. There was agreement that improvements in PPH detection were necessary, suggesting greater acceptance of implementing the calibrated drape element of the bundle. There were more discordant beliefs about the benefits of giving tranexamic acid, which is perhaps not surprising given that conclusive evidence of safety and effectiveness has only emerged in the past five years [34]. There were also persistent concerns expressed around the sustainability of the bundle and drapes, along with ongoing stock-outs of essential supplies and drugs and inadequate training.

This study aimed to inform and to support the implementation of the new E-MOTIVE bundle which will be tested in a clinical trial. Whilst it is widely evidenced that clinical bundles can standardise practice and patient care [35], there is also evidence that changing any clinical behaviours, including PPH-related detection and management, is likely to come with implementation challenges [14]. Therefore, in advance of the main trial, this formative research investigated what is likely to influence the implementation of the bundle and identify which implementation interventions could promote the use of the bundle in the contexts of Kenya, Nigeria, and South Africa. In addition to the previously reported influences on current PPH care, the TDF domain of intention was identified as a potential barrier to bundle implementation with participants stating that current management was deemed to be adequate, indicating that the implementation of the bundle might not give added value.

The Behaviour Change Wheel mapping enabled us to narrow the target of potential drivers of implementation and which types of implementation interventions would be best suited to address the identified barriers and enablers. The mapping to five intervention types indicated that the more relevant intervention types to improve PPH care were ‘education’ and ‘training’ to increase the understanding of using a bundle approach to PPH detection and management care and providing any new skills. Similarly, ‘persuasion’ through the communication of the benefits for healthcare providers by staff in a leadership role and ‘environmental restructuring’ including ‘enablement’ and ‘modelling’ to address the known barriers of delivering the bundle as intended. These intervention types were also identified as potential interventions to improve the implementation of other clinical care bundles, such as the ‘Sepsis Six’ [21].

### Strengths and limitations

This study focuses on factors influencing behaviours related to PPH detection and management and how they could pose challenges for the future implementation of a new clinical bundle. The inclusion of first responders—midwives, nurses, doctors and managers with varying experiences—did provide more insight into the challenges of coping with PPH and the future uptake of E-MOTIVE. However, the clinical position of the researchers conducting the interviews may have impacted the responses given about actual practice; for example, if a doctor interviewed a junior midwife, she might give an answer based on what she thought the doctor would want to hear, rather than on reflecting actual clinical practices (social desirability bias). Efforts to minimise this influence included training interviewers on appropriate probing and to avoid judgmental language including continuous monitoring of responses and feedback throughout the data collection period by the formative research team.

A number of barriers were identified, and many of which were structural- and resource-related, such as lack of electricity, staff and number of hospital beds. While we recognise the importance of these barriers, they are unfortunately outside the scope of the trial interventions to feasibly and sustainably address them. In the main trial process evaluation, we plan to document these contextual challenges and ensure that they are accounted for in future efforts to scale-up PPH detection and management.

Approaches to changing clinical behaviours tend to be pragmatic; therefore, demonstrating how improvements were achieved is more difficult in the absence of any theory-based development or conceptualisation. The major strengths of this study are the systematic and theoretical approach to specifying the types of interventions required using implementation and behavioural science methodologies. These behaviour change frameworks have been used across many clinical settings for identifying implementation problems and informing implementation interventions, but not yet in the PPH field. This approach is in keeping with the importance placed on early consideration of implementation to increase the development of an intervention (such as E-MOTIVE), as advocated by the United Kingdom Medical Research Council [36].

An issue that emerged from the study was how challenging participants found it to conceptualise using a ‘bundled’ approach of clinical care, as this was a new and hypothetical approach for most participants. To enhance understanding about the E-MOTIVE bundle, participants were shown pictures of the new calibrated drape and a pictograph of the bundle, and the bundled approach was described by the researcher conducting the interview. Despite this, the findings from anticipated, hypothetical use of the bundle may differ from those discovered from actual use. However, participants noted a number of potential issues based on current practice and what they would see as likely issues to using the bundle hypothetically, and what would help to implement it in their specific contexts. This hypothetical bundle implementation enables us to think about actual implementation from the outset of project development, rather than waiting to address potential implementation gaps or failures at the trial stage. The approach gives the bundle and interventions the best chance of succeeding and maximising the internal validity of the implementation trial by ensuring the bundle is delivered with fidelity and giving us the optimal opportunity of evaluating it. Barriers and enablers to the actual use of the E-MOTIVE bundle will be assessed as part of a process evaluation of the E-MOTIVE trial.

## Conclusions

To our knowledge, this is the first study to conduct theory-based formative research with healthcare providers of a new clinical bundle to inform the design of implementation interventions aiming to improve the detection and management of PPH in low-resource, high-burden settings. It provides additional insights into what implementation intervention might enhance efforts to create the desired changes in PPH detection and management behaviours to maximise bundle uptake. Despite, many participants expressing preliminary acceptance of adopting a bundled approach, some changes are necessary to promote and increase bundle uptake by addressing the identified challenges. These changes include training on using the E-MOTIVE bundle elements all together (rather than a wait-and-see sequential delivery), training new skills for midwives to administer IV tranexamic acid, promoting more teamwork and communication and ensuring an adequate of stock of the necessary consumables, equipment and drugs to deliver the bundle. These findings underscore a critical need to develop strategies to address implementation challenges whereby our formative research may maximise the uptake and appropriate use of the new E-MOTIVE bundle by basing the implementation intervention on the barriers from current practice and challenges identified by the potential users of the bundle.

The next step is further research during the E-MOTIVE clinical trial to investigate the acceptability and usability of these implementation interventions with healthcare providers and stakeholders in Nigeria, Kenya and South Africa.

## Supplementary Information

**Additional file 1.** Consolidated criteria for reporting qualitative studies (COREQ): 32-item checklist

**Additional file 2.** In-depth interview guide: healthcare providers

**Additional file 3.** Additional File 3 Full list of key barriers and enablers of PPH detection and management in Nigeria, Kenya and South Africa

## Data Availability

The datasets used and/or analysed during the current study are available from the corresponding author on reasonable request.

## Abbreviations

BCT: Behavioural Change Technique
E-MOTIVE: Early detection using a calibrated drape, Massage of uterus, administration of Oxytocic drugs, administration of Tranexamic acid, administration of IntraVenous fluids and Examine genital tract and escalate
LMIC: Low- and middle-income countries
PPH: Postpartum haemorrhage
TDF: Theoretical Domains Framework
WHO: World Health Organization
