# Supplementary material for: Factors influencing postpartum haemorrhage detection and management and the implementation of a new postpartum haemorrhage care bundle (E-MOTIVE) in Kenya, Nigeria, and South Africa
Source: Implement Sci. 2023 Jan 11;18:1. doi: 10.1186/s13012-022-01253-0 (PMC9832403; doi:10.1186/s13012-022-01253-0)
Supplement: Supplementary file 3 — Additional file 3. Additional File 3 Full list of key barriers and enablers of PPH detection and management in Nigeria, Kenya and South Africa [file 13012_2022_1253_MOESM3_ESM.docx]

Additional File 3 – Full list of key barriers and enablers of PPH detection and management in Nigeria, Kenya and South Africa

This table presents a full list of the beliefs statements and the overall frequency count of the number of participants who expressed the belief (n=maximum of 45 participants). Belief statements are classified as either a Barrier (B), Enabler (E) or Mixed (M) for each country.

| Key findings grouped by TDF Domain | Frequency (N=45) | Nigeria | Kenya | South Africa | Example Quote(s) |
| --- | --- | --- | --- | --- | --- |
| **Environmental Context and Resources** |  |  |  |  |  |
| Health hospitals organised into separate wards or rooms for maternity care | 45 | E | E | E | “It is made up of 2 wards, the labour room where we admit all the labour patients in, we have 5 beds in each ward” (Nurse, Nigeria) |
| Guidelines, clinical protocols, posters related to PPH are (not) displayed in the maternity ward | 41 | M | E | M | “We have SOPs in the labour ward and they are all useful. The protocols are displayed on the walls. Everyone can check quickly” (Nurse, Kenya)  “No … I don’t really know why they [posters, charts] are not displayed” (Nurse, South Africa) |
| Variable readiness of theatres and availability of surgeons | 29 | M | M | M | “We don’t have problem with them [theatres]actually, they run calls here at any point on time you have anaesthetic on call, the peri-op on call, and other staffs in the theatre so we only have to inform them” (Doctor, Nigeria)  “Have one theatre so there can be delays getting the patient into theatre if there is another emergency” (Doctor, South Africa) |
| Lack of availability of oxytocin, misoprostol and tranexamic acid in some settings | 26 | B | X | M | “Not available here like the Soxyn, Novartis [brands of oxytocin] is good but it is not available because is expensive actually” (Doctor, Nigeria)  “We've been having only oxytocin and misoprostol…. that’s what we've been using otherwise there was nothing” (Admin, South Africa) |
| Lack of reliable supply of blood from hospital blood bank | 20 | B | E | B | “Blood might not be available at the blood bank, we have to wait for the patients’ relatives” (Doctor, Nigeria)  “We have blood ready, in case transfusion is needed” (Nurse, Kenya) |
| Lack of staff available to manage PPH | 16 | B | B | B | “Sometimes it will be only two persons on a shift and you have maybe two patients that are having PPH and you have some of the patients on normal labour” (Midwife, Nigeria) |
| No appropriate tools available for blood loss estimation | 15 | B | B | X | “We normally don’t have instruments to quantify the blood loss so basically it is based on visual inspection” (Doctor, Nigeria) |
| Rely on woman’s relatives to procure tranexamic acid | 12 | B | X | X | “It is a challenge because sometimes the patient cannot afford [TXA] because of the cost” (Doctor, Nigeria) |
| Essential equipment (i.e., vaginal balloon tamponade) is not always available | 9 | B | X | M | “PPH box…. everything is together there is IV lines in there is catheters in it balloon tamponades in it, cyclokapron [TXA] so everything is there” (Midwife, South Africa)  “Let’s say uterine tamponade…. we can’t sustain then we can make our own improvised methods” (Doctor, Nigeria) |
| Varying bed capacity in hospital to cope with high volume of referred woman who are unbooked | 5 | X | B | X | “The referral hospital are usually full and may not have space for extra patients” (Midwife, Kenya) |
| Team-working is limited by a lack of staff and a lack of space within labour room or ward | 4 | X | X | B | “I think it can improve teamwork could you need more people” (Midwife, South Africa) |
| **Skills** |  |  |  |  |  |
| Inconsistent in-service training in PPH | 44 | M | M | M | “We do patient training, for example sometimes, at a time, there was a time when there was a demonstration on the use of anti-shock garment and all that, and most of the training are not online” (Doctor, Nigeria)  “There is not any other training outside medical school and residency training program specifically on postpartum haemorrhage” (Admin, Nigeria) |
| Regular continuing medical education (CME), including case reviews is available | 16 | E | E | E | “We also undertake CMEs, monthly or twice a month” (Midwife, Kenya) |
| Continuous and additional training helps to keep up-to-date with new approaches and current recommendations | 14 | X | E | E | “It is very much helpful because you cannot only rely on the knowledge from training school while there are new trends” (Midwife, Kenya) |
| Additional training required for less experienced or new staff who are likely to be first responders | 13 | B | B | E | “The only training, I will say I have received was when the department presented their protocol on the management of PPH. That was the only additional training I would say I have received” (Nurse, Nigeria)  “I think the [training] drills are important for young doctors to detect PPH” (Admin, South Africa) |
| Introducing hospital-specific guidelines and risk assessment protocols for PPH will reduce PPH cases | 5 | E | X | E | “We are following is the RCOG guidelines. But then still, the problem with that is that there are some things that cannot really fit into our environment, taking the peculiarity of our environment…. we bring out our own protocol which is more suited to our environment that is where we are going to be using it” (Doctor, Nigeria) |
| Multi-disciplinary simulation training perceived as more beneficial | 2 | X | X | E | “We're going to do this simultaneous everybody being there, being exposed at the same time, will learn it's sort of a fire drill for all of us” (Midwife, South Africa) |
| All staff may not have similar level of knowledge and expertise | 1 | X | X | B | “It also gets because now you have someone that is going run but they don’t know where, so knowledge gets [in the way]” (Doctor, South Africa) |
| Team-working hampered by variable knowledge and skills of staff | 1 | X | X | B | “if you are.... lucky to have people who have been trained for PPH. And mostly, most of the time people are not trained” (Admin, South Africa) |
| **Knowledge** |  |  |  |  |  |
| Variable understanding of how to detect different aetiologies of PPH | 44 | E | M | B | “Postpartum haemorrhage is by definition, a vaginal bleeding after birth. And in terms of quantification when a woman has lost more, more or equal 500 ml of blood at the definition” (Doctor, South Africa)  "I think it is not having enough knowledge [among new staff] on the PPH…Because maybe sometimes we are unable to quantify the amount of blood lost, then we say this is PPH, or this is just normal blood loss" (Doctor, Kenya) |
| Variable understanding of what constitutes appropriate clinical practice for PPH management | 37 | E | M | B | “We always have them be made aware of these guidelines. But for the rest of us, we [Doctors] sort of go ahead without knowing or reading the guidelines” (Head of Dept, South Africa)  “We always use them as a reference. We want all of us to have the same level of understanding and care” (Midwife, South Africa) |
| Higher-level hospitals receive limited information about the previous care received by woman referred from lower-hospitals | 23 | B | B | X | “In some cases, the patient arrives here with no IV line, no samples, and quite often the reason for referral could be something they could have easily managed, such as retained placenta, but they do not know what to do” (Nurse, Kenya) |
| Good awareness of the signs and symptoms of PPH | 17 | E | X | E | “Postpartum haemorrhage is when the woman delivered, in terms of vaginal birth, when she delivered and has a bleeding in excess of 500mls or any amount of bleeding that can make the woman, or any amount of bleeding that would make the woman have haemodynamic instability” (Doctor, Nigeria) |
| Views about healthcare providers needing to be aware of medicine stock levels | 7 | X | X | E | “It would be nice to know that we have stock and those things that we need to do those things are there” (Doctor, South Africa) |
| Limited knowledge about existing delays in PPH detection post birth and appropriate management of PPH | 3 | X | X | B | “We won't be misdiagnosing patients” (Midwife, South Africa) |
| Having the patient history would accelerate treatment | 1 | X | X | E | “Some of the patients are not even discussed before referral they came with no clinical notes and those are the difficult ones. You basically have to start from a scratch” (Doctor, South Africa). |
| **Behavioural Regulation** |  |  |  |  |  |
| Receiving feedback on current practice helps identify areas for improvement | 38 | E | E | E | “I think anything that could make it better, it’s still welcomed” (Doctor, Nigeria) |
| Have or do not have quality improvement strategies for PPH (e.g., meetings where feedback is given, having a PPH kit, continuing education and hospital specific protocols are perceived to work) | 17 | M | M | M | “We would like to hold such meetings more frequently. Sometimes due to inadequate staffing we skip monthly meetings” (Midwife, Kenya)  “[receive feedback] from the doctor that has helped us treat the patient” (Midwife, South Africa) |
| Current strategies in place, e.g., having a PPH kit, continuing education and hospital specific protocols are perceived to work | 16 | X | E | E | “I am not aware of any specific strategies of improvement, apart from perhaps resuming CMEs, whereby clinicians would continue to get training on PPH management” (Doctor, Kenya) |
| **Beliefs about Consequences** |  |  |  |  |  |
| Maternal mortality due to PPH is low if birth occurs within hospital’ | 38 | B | M | M | “It is really it’s a big issue especially with those that are referred from another hospital or referred from home...… [if] it occurs in our centre, because of early intervention and prompt treatment, we usually have good outcome” (Doctor, Nigeria)  “It’s very common we see it very often, but I think we respond to it quite quickly .... within an hour or two after birth…. so, every PPH will get picked up at a certain” (Midwife, South Africa) |
| Lack of accurate and objective measurement of blood loss delays detection of PPH | 34 | B | B | B | “We still rely on the gross method that we use, of assessing the amount of blood based on the amount of blood by our under-pads or the sanitary pads that we use or occasionally if it is too much, we use the kidney dish to collect the blood but you know we have a high tendency for underestimating because it will not take into account” (Admin, Nigeria) |
| Referrals by other hospitals can be too late for the receiving hospital to effectively manage the PPH | 21 | B | B | B | “Late presentation is also one of the issues and challenges that we have especially from those who are coming from outside because most times they would have gone to other peripheral hospitals” (Doctor, Nigeria) |
| **Emotion** |  |  |  |  |  |
| Negative emotions resulting from the unpredictability and fatality associated with PPH | 40 | M | M | M | “You are a little bit on edge you know that this is a complication it’s a leading cause of maternal mortality” (Midwife, South Africa)  “it’s an incident that can be managed. So, it’s just that nothing to fear or to worry about” (Doctor, Nigeria) |
| Limited impact from emotions as doctors’ work ‘on autopilot’ | 7 | E | E | X | “At that time, you are being put on one autopilot that all you're trying to do is trying to save that person’s life, so even thinking of yourself as being stressed out does not even come into play, it’s after you’ve finished” (Doctor, Nigeria) |
| Emotional support is received from colleagues and religious beliefs | 7 | E | E | X | “There is need for debriefs, especially after a complication ends in maternal death” (Midwife, Nigeria) |
| Worry about being threatened and blamed by supervisors or by families for any bad outcome | 1 | X | X | B | “I’m always concerned when there is a complaint [from the community]” (Midwife, South Africa) |
| **Social Influences** |  |  |  |  |  |
| Team-working is necessary to manage a PPH | 42 | E | E | E | “We worked with the midwife who conducted the delivery and also some other nurses and me as a Medical Officer” (Doctor, Kenya) |
| Team-working reassures individuals that support is readily available | 8 | E | E | E | “We work well as a team and when you call for the help everyone comes here. Everyone comes here, everyone doctors, nurses, even the cleaners at times” (Midwife, South Africa) |
| Post giving birth, mothers could not be aware of the PPH signs and symptoms | 3 | X | X | B | “I’m bleeding too much, so you educate your patient please call for help as much as bleeding after delivery is normal but excessive bleeding is not normal” (Midwife, South Africa) |
| **Memory, Attention and Decision-Making** |  |  |  |  |  |
| International guidelines adapted into hospital-specific clinical guidelines are more likely to be implemented by staff | 36 | M | M | M | “We use WHO guidelines. But we have tailored these to the basics. We sometimes substitute the drugs of choice as per the guidelines, with what we have available in the hospital” (Doctor, Kenya)  “We [Doctors] sort of go ahead without knowing or reading the guidelines” (Head of Dept, South Africa) |
| Good clinical understanding of when and how to escalate to refractory PPH management intervention | 24 | M | E | M | “Patient can be taken to a theatre…. under resuscitation on IV fluids and blood if available, check for cervical laceration, and then since the uterus is flabby has not contracted, we can institute other modalities like minimally invasive modalities or more extensive modalities” (Doctor, Nigeria)  “Oxytocic was given, then checking if the uterus has contracted yet and then he noticed that the uterus was not contracting and he did the bimanual contraction of the uterus until the doctor came” (Midwife, South Africa) |
| Delayed treatment due to reliance on vital signs to detect a PPH | 10 | M | B | M | “Especially from the signs and symptoms the mother starts experiencing headaches, light headedness and also dizziness and from the vitals when you check the blood pressure” (Doctor, Kenya)  “The extent of blood loss is the first thing that we pay attention to, and then the patient’s vital status” (Doctor, Nigeria) |
| Standardised methods to detect PPH would improve PPH management | 3 | X | X | E | “The benefit of the patient……. if we treat each patient with PPH in a standard in a standard way” (Midwife, South Africa) |
| **Social/Professional Role and Identity** |  |  |  |  |  |
| Good levels of role clarity | 34 | E | E | E | “My role is that after I detect that the woman has PPH, I will call the attention of my colleague and later on the doctor on call” (Midwife, Nigeria) |
| Restrictions as to which professional roles are allowed to perform certain parts of the bundle | 33 | B | B | B | “The person doing the delivery, the midwife will examine and then they would call, usually call the doctor who take over examination or just double check” (Midwife, South Africa) |
| The team managing a PPH should be multi-disciplinary | 10 | X | E | X | “Midwives even the sub-staff can help but if there are many doctors on ground they can come and help in managing the patient. For instance, one setting the line, one taking the sample for blood for grouping and cross-matching or some applying anti-shock garment, so it is multi-disciplinary activity” (Doctor, Kenya) |
| **Beliefs about Capabilities** |  |  |  |  |  |
| Varied levels of confidence in ability to detect and manage PPH | 24 | M | M | M | “I can confidently say I can be able to do it” (Midwife, South Africa)  “Not all workers, like nurses and intern doctors [are confident]” (Doctor, Kenya) |
| Concerns about abilities to detect and manage PPH with un-booked women | 6 | B | X | X | “The patients come from outside (un-booked) because that's where the problem is” (Admin, South Africa) |
| Coping with the quick and sudden onset of PPH can be challenging | 2 | B | X | X | “Her condition suddenly changed and she showed symptoms of PPH” (Midwife, Kenya) |
| Limited capacity of other hospitals to provide appropriate initial treatment before referring women | 2 | X | X | B | “They [referring hospital] said a patient she is PPH but the patient doesn't have catheter, has only got one line….” (Midwife, South Africa) |
| **Reinforcement** |  |  |  |  |  |
| Varied approaches to disciplinary procedures across hospitals and countries | 29 | E | B | B | “When something goes wrong, the entire team should take responsibility, review what happened and use it positively as a learning experience” (Doctor, Kenya)  “If there was a higher incidents of obstetric haemorrhage ……they would either give training or they would discipline the people involved” (Midwife, South Africa) |
| Disciplinary procedures across hospitals and countries are unlikely to change PPH care | 18 | B | B | X | “The most important thing is about having the passion…. [to]save the patient rather than thinking that whether you are going to be disciplined for doing it or not” (Admin, Nigeria) |
| Fear of punishment for failing to detect or manage a PPH | 1 | X | X | B | “We are always anxious about mismanaging the patient and the patient will end up dead or something” [mismanagement could result in disciplinary action]” (Midwife, South Africa) |
| **Goals** |  |  |  |  |  |
| Mortality associated with PPH makes it a clinical priority | 42 | E | E | E | “It’s a very huge priority because for me it’s a very serious challenge and very serious priority that I need to consider it with too much seriousness. Because it’s something that I may just…can result to maternal mortality within the blink of an eye” (Midwife, Nigeria) |
| Eclampsia perceived as a higher priority than PPH because eclampsia causes more mortality | 3 | B | B | B | “Severe pre-eclampsia and eclampsia are first on priority list as they cause more mortality” (Doctor, Nigeria) |

X=belief statement not identified in the data collected from the country
